# Electrocatalytic Detection of Amitrole on the Multi-Walled Carbon Nanotube – Iron (II) tetra-aminophthalocyanine Platform

**DOI:** 10.3390/s8085096

**Published:** 2008-08-27

**Authors:** Msimelelo Siswana, Kenneth I. Ozoemena, Tebello Nyokong

**Affiliations:** 1 Department of Chemistry, Rhodes University, Grahamstown 6140, South Africa; E-Mail: msiswana@wsu.ac.za (M.S.); T.Nyokong@ru.ac.za (T.N); 2 Molecular and Nanomaterials Electrochemistry Laboratory, Department of Chemistry, University of Pretoria, Pretoria 0002, South Africa

**Keywords:** Amitrole, tetra-aminophthalocyanine, electropolymerization, multi walled carbon nanotubes, basal plane pyrolytic graphite electrode

## Abstract

It is shown that iron(II) tetra-aminophthalocyanine complex electropolymerized onto a multi-walled carbon nanotube-modified basal plane pyrolytic graphite electrode greatly enhanced the electrocatalytic detetion of amitrole (a toxic herbicide), resulting in a very low detection limit (0.5 nM) and excellent sensitivity of 8.80±0.44 μA/nM, compared to any known work reported so far. The electrocatalytic detection of amitrole at this electrode occurred at less positive potential (∼0.3 V vs Ag|ACl) and also revealed a typical coupled chemical reaction. The mechanism for this response is proposed. The electrode gave satisfactory selectivity to amitrole in the presence of other potential interfering pesticides in aqueous solutions.

## 1. Introduction

**Figure 1.**
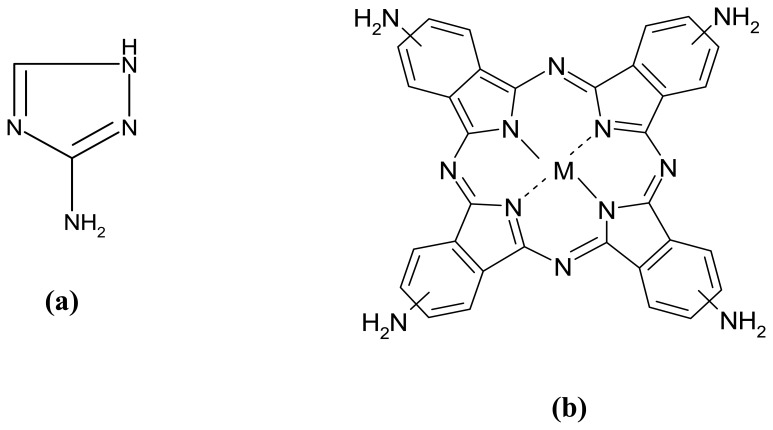
Molecular structures of (**a**) amitrole and (**b**) metal tetra-aminophthalocyanine (MTAPc, where M = Fe, Co, Mn and Ni).

Amitrole (3-amino-1,2,4-triazole, Figure 1a) is a toxic non-selective herbicide used to control weeds in agriculture and along railway tracks [1]. In 1971, the use of this herbicide for food crops was cancelled by the Environmental Protection Agency (EPA) of the United States of America [2]. Due to its toxicity and high solubility in water this herbicide has a low threshold limit value of 0.1 μg/L (*ca.* 1 nM), set by the European Economic Commission (EEC) directive [3]. To our knowledge, the best electroanalytical response (in terms of sensitivity and limit of detection) so far for amitrole was with carbon paste electrode modified with the nano-scaled iron(II)phthalocyanine, where a detection limit of 0.305 μg/L and sensitivity of 3.44 μA/nM were obtained at 0.42 V vs Ag|AgCl [4]. Therefore, the search for highly sensitive methods for the detection of amitrole, and especially in Africa, where there is no accessible document for its regulation as herbicide in agriculture, is essential.

Electrochemical detection of toxic organic materials with modified carbon electrodes has held the interest of electrochemists and other scientists for many years. Transition metallophthalocyanine complexes have proved themselves as excellent electrocatalysts for the detection of organic complexes of environmental, biomedical and industrial importance [5, 6]. Carbon nanotube modified electrodes have continued to receive significant attention in electroanalysis [6-8]. Smart integration of carbon nanotubes with metallophthalocyanine complexes on electrode surfaces has the potential to improve the electrocatalytic response of certain analytes [5, 9-11].

In this communication, we explore the electrocatalytic response of amitrole at a basal plane pyrolytic graphite electrode pre-modified with acid-treated MWCNTs and then electro-coated with the polymer of iron (II) tetra-aminophthalocyanine complex (FeTAPc, Figure 1b). For the first time, we show that this carbon nanotube-metallophthalocyanine hybrid greatly enhances the electrocatalytic response of amitrole in the sub-nanomolar concentrations.

## 2. Results and Discussion

### 2.1 Comparative Cyclic Voltammetric Responses Towards Amitrole

**Figure 2.**
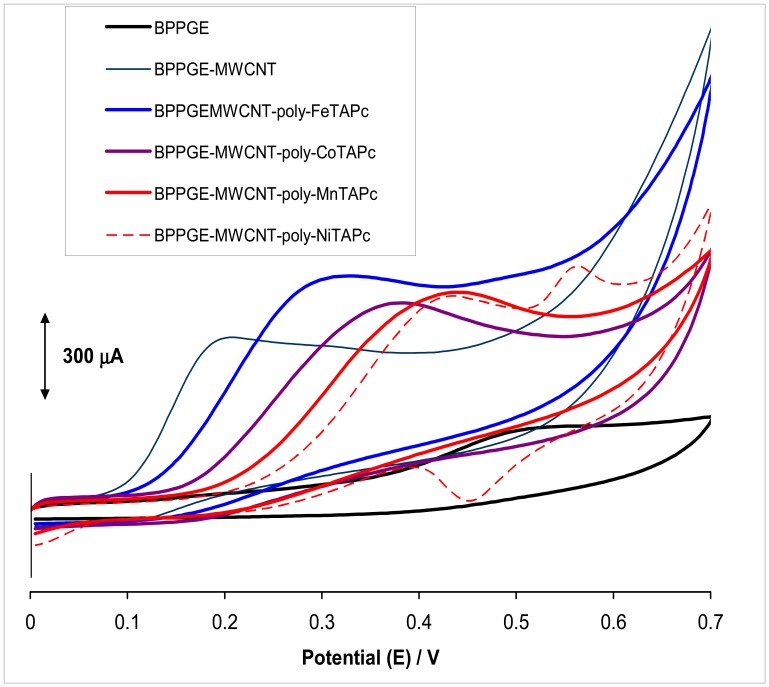
Comparative cyclic voltammograms of 1 mM amitrole in 0.1 M phosphate buffer, pH 12 on bare and modified BPPGEs. Supporting electrolyte is pH 12 phosphate buffer containing 0.05 M Na_2_SO_4_. Scan rate = 25 mV.s^-1^.

Figure 2 shows the oxidation of amitrole on BPPGE, MWCNT-BPPGE, and MTAPc-MWCNT-BPPGE (M = Fe, Co, Mn or Ni). The most important observations obtained from the graphs are: (i) there is a broad and weak peak due to oxidation of amitrole on unmodified BPPGE; (ii) the peak due to oxidation of amitrole is much larger on both BPPGE-MWCNT and BPPGE- MWCNT-*poly*-MTAPc and is observed at less positive potentials on the former showing that the former is a better catalyst than the MTAPc complexes for the oxidation of amitrole.

However, larger currents are observed on MTAPc compared to MWCNT-BPPGE; (iii) amongst the metal tetraaminophthalocyanine-modified electrodes, the catalytic efficiency (judged by shifting of the oxidation potential for amitrole to less positive values) towards amitrole oxidation follows the order: FeTAPc > CoTAPc > MnTAPc > NiTAPc. It is well known that metallophthalocyanine complexes containing Fe, Mn and Co as central metals are good catalysts for many reactions. However, this complex shows poor catalytic activity for NiTAPc when compared to FeTAPc, MnTAPc and CoTAPc. Since BPPGE-MWCNT-*poly*-FeTAPc gave the highest electrocatalytic response compared to the other electrodes, all subsequent studies were carried out with this FeTAPc-based electrode.

### 2.2 Effect of Varying Scan Rates

**Figure 3.**
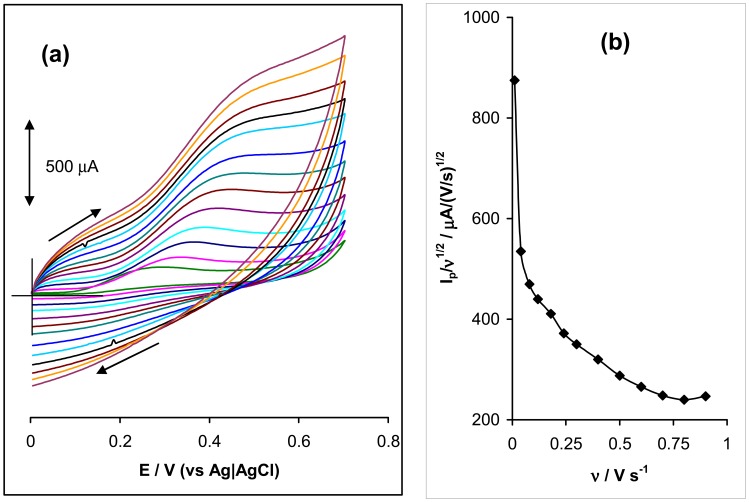
**(a)** Examples of cyclc voltammetric evolutions of BPPGE-MWCNT-poly-FeTAPc obtained in 0.1 M phosphate buffer (pH 12) containing 1 mM amitrole at scan rates 10, 40, 80, 120, 160, 240, 300, 400, 500, 600, 700, 800 and 900 mVs^-1^ (inner to outer). **(b)** Current function plot, I_p_/ν ^1/2^ vs ν.

Figure 3 shows the voltammetric evolution of the BPPGE-MWCNT-*poly*-FeTAPc at scan rates ranging from 10 – 900 mVs^-1^ in a pH 12.0 solution containing 10^-3^ M amitrole. We observed linear variation of the peak current with the square root of scan rate (ν ^1/2^) (R^2^ = 0.9933) (not shown).

This result clearly indicates a diffusion-controlled electro-oxidative process. The plot stabilises from > 300 mVs^-1^ (not shown) Also, the peak current varied linearly with the scan rate (ν) (R^2^ = 0.9900) from 10 – 180 mVs^-1^, stabilising from > 200 mVs^-1^ (not shown). Since this type of process is characteristic of surface-adsorbed electrochemical process; it suggests the likelihood of amitrole or oxidation products and/or intermediate(s) also adsorbing onto the electrode surface. Interesting, the current function plot (Figure 3b) gave the characteristic shape of a coupled chemical reaction (EC_cat_) for the amitrole, clearly confirming electrocatalytic activity. On the basis of the information, we suggest the following mechanism for the oxidation of amitrole. The redox process of the MWCNT-confined FeTAPc species is:

(1) $$({\text{Fe}}^{3+}\text{TAPc})\text{film}⥬({\text{Fe}}^{2+}\text{TAPc})\text{film}$$

The interaction of the Fe^3+^TAPc with aqueous amitrole results to the regeneration of the Fe^2+^TAPc and the formation of amitrole oxidation intermediates:

(2) $$\left({\text{Fe}}^{3+}\text{TAPc})+\text{Amitrole}\;(\text{aq})\to ({\text{Fe}}^{3+}\text{TAPc})\text{film}\;(\text{Amitrole}\right)$$

(3) $$({\text{Fe}}^{3+}\text{TAPc})\text{film}\;(\text{Amitrole})\to ({\text{Fe}}^{2+}\text{TAPc})\text{film}+\text{Intermediates}\;+\text{e}-$$

The intermediate(s) also coordinate with Fe^3+^TAPc film and are further oxidized to the final product(s), via similar mediated electro-oxidation process:

(4) $$\left({\text{Fe}}^{3+}\text{TAPc})\text{film}+\text{Intermediates}\to ({\text{Fe}}^{3+}\text{TAPc})\text{film}\;(\text{Intermediates}\right)$$

(5) $$({\text{Fe}}^{3+}\text{TAPc})\text{film}\;(\text{Intermediates})\to ({\text{Fe}}^{2+}\text{TAPc})\text{film}+\text{Products}$$

We are not aware of the electrooxidative product(s) of amitrole, and it is important that this be pursued in future work.

### 2.3 Chronoamperometric Studies: Analytical Utility

**Figure 4.**
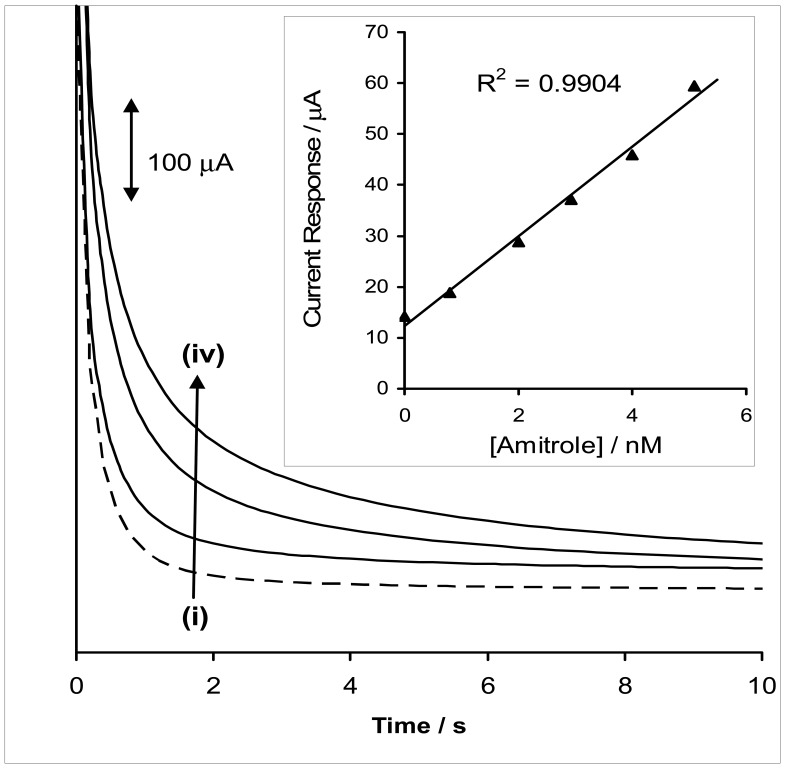
Examples of chronoamperometric evolutions of the BPPGE-MWCNT-poly-FeTAPc obtained in 0.1 M phosphate buffer (pH 12) containing different concentrations of amitrole (0.0, 3.0, 4.0 and 5.0 nM (from (i) to (iv)) at fixed potential of 0.4 V vs Ag|AgCl. Inset is a typical plot of current response vs concentration of amitrole.

Figure 4 shows the chronoamperomegrams recorded at different concentrations of amitrole at a fixed potential E = 0.4 V vs Ag|AgCl. Since the oxidation of amitrole could lead to formation of radicals/intermediates (as already discussed in the scan rate studies above) that could readily combine with the polymer film and poison the electrode, the electrode surface was conditioned between measurements by rinsing in buffer (pH 12).

We observed that more than 96 % of the original current of each concentration of amitrole analysed is regenerated after this treatment, suggesting the viability of continuous use of this electrode once fabricated. A linear relationship between current response and amitrole concentrations was obtained (using LINEST statistical programme) as:

(6) $$\begin{array}{cc} {\text{I}}_{\text{p}}/\mu \text{A}=(8.80\pm 0.44)\;\left[\text{Amitrole}\right]/\text{nM}+(12.35\pm 2.35) & \left({\text{R}}^{2}=0.9904\right) \end{array}$$

The limit of detection (LoD = 3.3 s/m [12], where *s* is the relative standard deviation of the intercept and *m* the slope of the linear current versus the concentration of amitrole) was calculated as 0.493 nM. When compared to the presently available literature reports on the electrocatalytic detection of amitrole [13-16], this report gave the best analytical response in terms of: (i) less positive detection potential (0.3 V vs Ag|AgCl); (ii) low detection limit (approximately 50% lower than the regulatory limit), and (iii) high sensitivity (approximately two and half times higher than the 3.44 μA/nM reported for carbon paste electrode modified with iron (II) phthalocyanine nanoparticles [4]. The enhanced sensitivity obtained in this work is attributed to the ability of multi-walled carbon nanotubes to act as efficient conducting species for iron(II) tetra-aminophthalocyanine electrocatalyst.

### 2.4 Selectivity Studies

**Figure 5.**
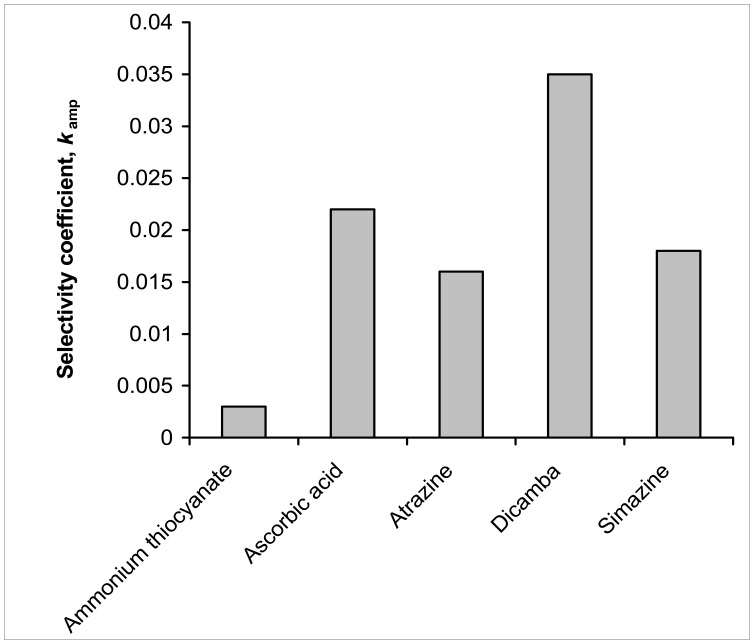
Amperometric selectivity coefficient of different potential interfering species in the presence of amitrole.

The selectivity of the FeTAPc-modified BPPGE-MWCNT was investigated using the mixed solution method [17]. The concentration of the interfering species and amitrole were 10^-6^ and 10^-7^ M, respectively. The selectivity was checked against the pesticides atrazine, simazine, 3,6-dichloro-2-methoxybenzoic acid (dicamba) and ammonium thiocyanate (components of many herbicide formulations). The values of *K_amp_* (where *K_amp_* is the amperometric selectivity coefficient) were determined from Equation (7) for analysis in the presence of interfering ions:

(7) $${\text{K}}_{\text{amp}}=\left(\frac{\Delta {\text{I}}_{\text{mixture}}}{\Delta {\text{I}}_{\text{amitrole}}}-1\right)\frac{\left[\text{amitrole}\right]}{\left[\text{interferent}\right]}$$

where Δ*I_mixture_* and Δ*I_amitrole_* are respectively, the changes in current for the mixture containing amitrole and the interfering ion, and amitrole alone. The *K_amp_* values are graphically represented in Figure 5.

A K_amp_ value of less than 10^-3^ indicates non-interference, while one which falls within the order of 10^-3^ suggests that the species is an interferent, but not a strong one. Of all the possible interferents studied, dicamba interfered more strongly, while ammonium thiocyanate is the least interferent. Thus, the electrode could be most conveneinetly used for the detection of amitrole in solution where ammonium thiocyanate is present.

## 3. Experimental Section

Amitrole was obtained from Sigma. Iron(II) tetraaminophthalocyanine and (FeTAPc) and its related cobalt (CoTAPc), nickel (NiTAPc) and manganese (MnTAPc) counterparts were synthesized and characterized according to an established procedure [18]. Multi-walled carbon nanotubes (MWCNTs) were obtained from Nanolab. The basal plane pyrolytic graphite (BPPG) from which the basal plane pyrolytic graphite electrode (BPPGE) was constructed in-house, was obtained from Le Carbone (Sussex, UK). Norton caborundum paper (p1200 c) used to clean the electrode was purchased from Saint Gobain Abrasives (Saint-Gobain Abrasives (pty) Ltd., Isando, South Africa). Ultrapure water of resistivity 18.2 Ω was obtained from Milli-Q Water Systems (Millipore Corp., Bedford, MA, USA) and was used throughout for the preparation of solutions. Phosphate buffer solutions (PBS) were prepared with appropriate amounts of Na_2_HPO_4_ and NaH_2_PO_4_, and the pH adjusted with 0.1 M H_3_PO_4_ or NaOH. Salts such as NaCl, Na_2_SO_4_, CH3COONa, NaNO_3_ and NaClO_4_ were added to the buffer to test their effects on the redox peaks. All other reagents were of analytical grade and were used as received without further purification.

### 3.1 Apparatus

All electrochemical experiments were performed with an Autolab PGSTAT 30 potentiostat (Eco Chemie, Utrecht, The Netherlands) driven by the General Purpose Electrochemical Systems data processing software (GPES, software version 4.9). Chronoamperograms were obtained at 0.4 V (for FeTAPc), 0.5 V (for CoTAPc), 0.5 V (for MnTAPc), and 0.65 V (for NiTAPc). A conventional three-electrode system was used. The basal plane pyrolytic graphite electrode (BPPGE) disk (d = 5 mm in Teflon) used as working electrode was fabricated in-house by the Rhodes University Chemistry Machinery Workshop. Electrical contact with the disk was obtained via an inserted copper wire held in place with conducting silver varnish L 100 (Kemo^®^ Electronic, Germany). The working electrode was BPPGE modified with MWCNTs (BPPGE-MWCNT) or BPPGE modified with MWCNTs and Fe, Co, Mn or Ni tetra-aminophthalocyanine by electropolymerization (designated as BPPGE-MWCNT-*poly*-FeTAPc, BPPGE-MWCNT-*poly*-CoTAPc, BPPGE-MWCNT-*poly*-MnTAPc and BPPGE-MWCNT-*poly*-NiTAPc, respectively). A Ag|AgCl wire and platinum wire were used as pseudo-reference and counter electrodes, respectively. The potential response of Ag|AgCl pseudo-reference in aqueous conditions was less than the normal Ag|AgCl (3M KCl) and SCE by 0.15 V and ∼ 0.01 V, respectively. A Wissenschaftlick-Technische Werkstätten (WTW) pH 330/set-1 (Germany) pH meter was used for pH measurements. All solutions were deaerated by bubbling nitrogen prior to each electrochemical experiment. All experiments were performed at 25±1 °C.

### 3.2 Electrode modification

The MWCNTs were purified as described previously [19]. Briefly, MWCNTs (1 g) were added to 2.6 M HNO_3_ (140 mL) and the mixture was refluxed for 45 hours. The carbon nanotube sediment was separated from solution and washed with distilled water. It was then sonicated in a 3:1 mixture of H_2_SO_4_ and HNO_3_ for 24 hours. The sediment was thereafter washed with distilled water, stirred for 30 minutes in a 4:1 H_2_SO_4_/H_2_O_2_ mixture at 70°C, and washed again with distilled water. The purified MWCNT paste was then air-dried for 48 hours.

A BPPGE was prepared for use or for further modification by renewing the electrode surface with cellotape. This procedure involves polishing an old BPPGE surface on carborundum paper, pressing cellotape on the cleaned BPPGE surface and then removing the tape, along with several layers of graphite. Before use, the electrode was then rinsed in acetone to remove any adhesive. To prepare a carbon nanotube modified BPPGE (BPPGE-MWCNT) electrode, carbon nanotubes were abrasively immobilized onto the BPPGE by gently rubbing the electrode on a fine quality filter paper containing the carbon nanotubes [20]. To prepare a MWCNT-BPPGE modified with iron or cobalt or manganese or nickel tetra-amino phthalocyanine (FeTAPc, CoTAPc, MnTAPc or NiTAPc), the electropolymerization method was used. Briefly, the MWCNT-BPPGE was immersed in a ∼1 × 10^-3^ M solution of FeTAPc, CoTAPc, MnTAPc or NiTAPc in pure dry dimethylsulfoxide (DMSO) containing 10^-1^ M tetrabutylammonium tetrafluoroborate (TBABF_4_) as the supporting electrolyte and the solution was scanned within a potential range of -1.0 V and 1.2 V (vs Ag|AgCl) using cyclic voltammetry. The electrodes (BPPGE-MWCNT-*poly*-FeTAPc, BPPGE-MWCNT-*poly*-CoTAPc, BPPGE-MWCNT-*poly*-MnTAPc and BPPGE-MWCNT-*poly*-NiTAPc) were conditioned for use by immersing them in a 0.1 M phosphate buffer solutions of pH 12, and then scanning between -1.0 V and 1.0 V (vs Ag|AgCl). Ten cycles were sufficient to obtain stable cyclic voltammograms for all electrodes.

## 4. Conclusions

We have shown in this work that FeTAPc polymerized onto MWCNT-modified BPPGE can greatly enhance the electrocatalytic detection of amitrole at less positive potential, to about 0.5 nM level (which is approximately 50% lower than the regulatory limit of 1 nM), and with excellent sensitivity 8.8 μA/nM (more than 250% higher than the best report so far on elelctrocatalytic detection of amitrole using any metallophthalocyanine-based electrodes).
